# Characterization of rice farming systems, production constraints and determinants of adoption of improved varieties by smallholder farmers of the Republic of Benin

**DOI:** 10.1038/s41598-022-07946-2

**Published:** 2022-03-10

**Authors:** Yêyinou Laura Estelle Loko, Charlemagne D. S. J. Gbemavo, Gustave Djedatin, Eben-Ezer Ewedje, Azize Orobiyi, Joelle Toffa, Cyrille Tchakpa, Paulin Sedah, François Sabot

**Affiliations:** 1 National Higher School of Applied Biosciences and Biotechnologies (ENSBBA), National University of Sciences, Technologies, Engineering and Mathematics (UNSTIM), BP 14, Dassa-Zoumé, Benin; 2 DIADE UMR IRD/UM–Centre IRD de Montpellier, 911 av Agropolis, BP 604501, 34 394 Montpellier Cedex 5, France; 3 Jeune Équipe Associée À L’IRD (JEAI-GRAB), Dassa-Zoumé, Benin

**Keywords:** Agroecology, Environmental social sciences

## Abstract

The identification of technological and policy interventions allowing to improve the performance of Beninese rice systems is necessary to reduce the heavy dependence on rice imports. This study characterized the Beninese rice farming systems, identified the production constraints, and determinants of the adoption of improved varieties by farmers. Four hundred eighteen rice farm households were surveyed across 39 villages using participatory research tools and methods. Cluster analysis was used to classify the surveyed farm households and revealed four typologies of rice farming systems differentiated by 8 variables. These are, the intensive rice farming system (cluster 4; 33.7%), semi-intensive rice farming system (cluster 1; 31.8%), integrated rice–livestock farming system (cluster 3; 11.8%), and subsistence rice farming (cluster 2; 22.7%). The integrated rice–livestock farming system was the dominant type practiced in the northern Benin, while, it is the intensive rice farming system in the south. Fifteen production constraints across rice-growing areas were recorded. Our results suggest that to increase adoption of improved rice varieties, agricultural extension services should target landowners’ farmers practicing off-season rice production, and having other sources of income. Initiatives to boost rice production in Benin should prioritize the establishment of formal agricultural credit and mechanization option policies.

## Introduction

Rice is a cereal that strongly contributes to food security in the Republic of Benin with an estimated production of 406,000 tonnes in 2019^1^. However, the demand of rice from the Beninese populations is greater than its production, which leads to a high import estimated at 875,962 tonnes of rice and products in 2020^1^. Although Benin's rice yield (39,353 hg/ha) in 2020 was higher than the African average (22,061 hg/ha), it is far lower than that of Mauritania (52,703 hg/ha), the best West African producer^1^. This low yield is partially due to the various biotic and abiotic constraints encountered by Beninese farmers in rice production, as shown by previous^2–5^. However, these studies were restricted to a few districts and generally focused only on constraints found in irrigated rice production system. While, it is important to have a global view of the rice constraints and their variations across all production areas to find appropriate solutions boosting rice production in Republic of Benin.

In the Republic of Benin, smallholder farmers without financial means practice a subsistence rice cultivation^6^, which influences rice yields through the cultural practices such as fallow residue management, ploughing method and fertiliser use^4^. In addition, smallholder farmers apply various types of rice production systems, which affects also the performance and the potential of rice production^7^. It is therefore important to better characterise rice production systems in order to provide decision-makers and researchers with basic information for the implementation of measures to improve its production. Indeed, a good knowledge of farming systems is vital for the generation and application of appropriate technologies, to optimize the different stages of production and to contribute to improve farmers’ incomes^7,8^.

In the Republic of Benin, the dissemination of high-yielding rice varieties has accelerated in order to increase yield, in response to growing demand for this cereal^6^. Indeed, several improved rice varieties were introduced in traditional Beninese agriculture, with the IR841 variety as the most popular^9^. The improved rice varieties are known to positively influence productivity, therefore farmers' income and food security^10^. However, the released improved varieties do not fully meet the expectations of farmers and consumers^11^, which lead to numerous varietals dis-adoption^9^. Therefore, it is important to identify factors influencing this adoption across the main rice-growing areas of the country. Few studies on the determinants of the adoption of new technologies in agricultural sectors were done in the Republic of Benin. They are of paramount importance for initiating agricultural development because they make it possible to act on the key indicators identified to increase the probability of adopting new technologies. The literature provide very few information on this crucial information with regard to the rice sector in Benin despite its place of choice in improving the level of poverty in rural areas. Current studies focused mainly on determinants of adoption of NERICA (NEw RICe for Africa) varieties in some municipalities of central region^6,12^. However, a good understanding of the determinants of the adoption of improved rice varieties at the national level will allow developing effective strategies taking into account the regional differences. Indeed, adoption of improved rice varieties is important for increasing rice productivity and improving the living standard of the farmers in developing countries^13^.

This study aims to target technological and policy interventions permitting to improve the performance of rice systems and identify factors associated with the farmers’ adoption of improved rice varieties in order to boost rice production in the Republic of Benin. The specific objectives of this study were therefore to: (i) Characterise rice farming systems in the Republic of Benin; (ii) Identify rice production constraints and its variation throughout main rice growing areas; (iii) Identify determinants of adoption of rice-improved varieties by farmers in the study area.

## Methods

### Study area and sampling

**Figure 1:**
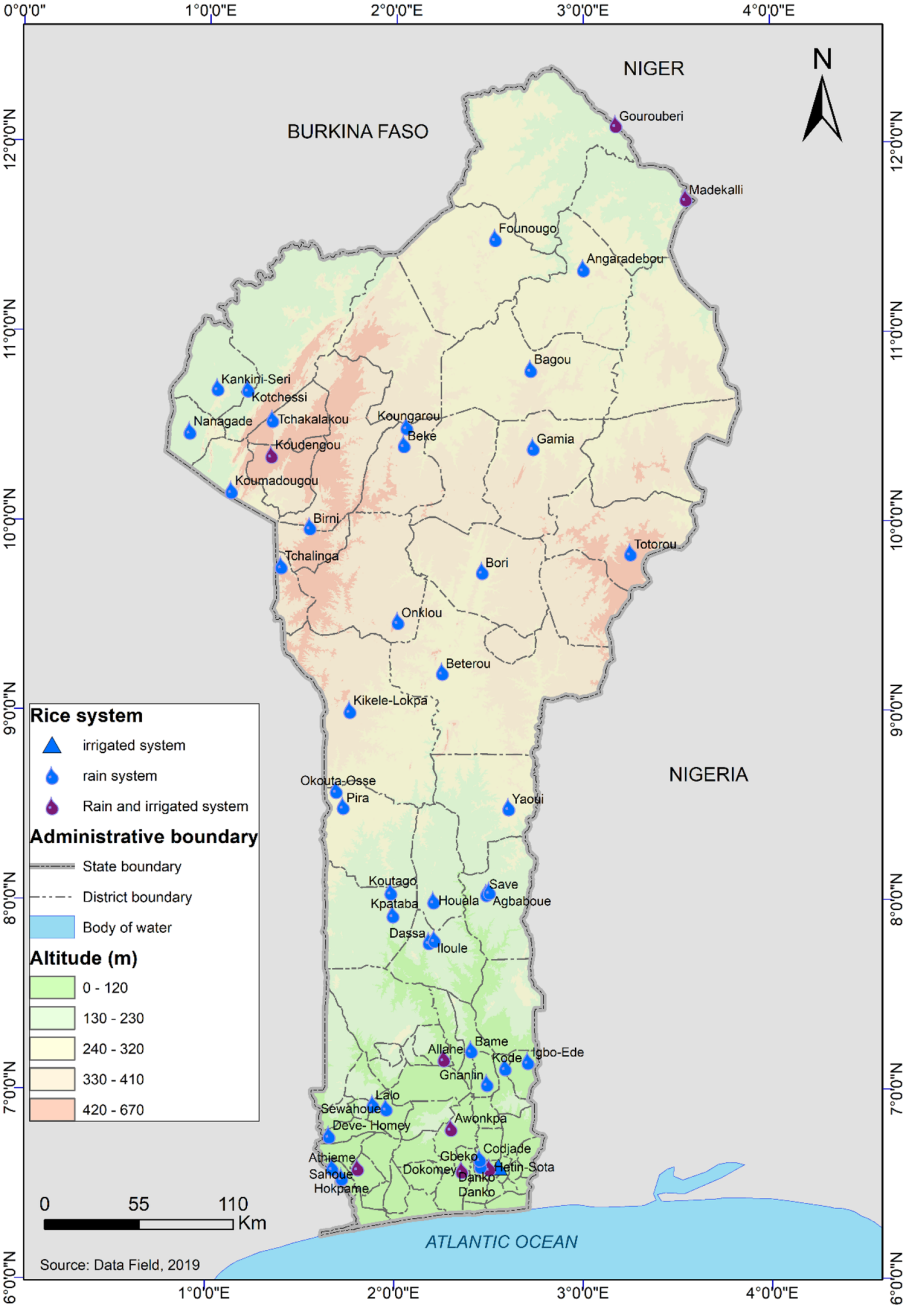
Map of the Republic of Benin showing the 39 surveyed villages and rice production systems in function of altitude in the study area. The figure was created using QGIS 3.10.13 software (www.qgis.org).

The studied population is located in the Republic of Benin, in the three climatic zones: Guineo Congolean zone (6°25′–7°30′N) in the south, Sudano-Guinean transition zone (7°30′–9°45′N) in the centre and Sudanian zone (9°45′–12°25′N) in the North. Indeed, rice is produced throughout the Beninese territory. The number of rice farmers to be surveyed was determine using the normal approximation of the binomial distribution^14^ (Eq. 1):

1 $$n=\frac{{U}_{1-\propto /2}^{2}\times p\left(1-p\right)}{{d}^{2}}$$

where n is the number of surveyed rice farmers; ${U}_{1-\propto /2}^{2}$ = 1.96 is the quantile of a standard normal distribution for a probability value of 0.05; *p* = 0.11 is the proportion of rice producers population; and d is the expected error margin of any parameter to be computed from the survey. For the present study the expected error margin (d) is fixed at 0.03 (this value is close to zero to have an accurate estimate of the parameters). The value of *p* was determine according to Adebo et al.^15^ by considering a single person interviewed per household, the number of agricultural households in the Republic of Benin (651,067 agricultural households)^16^, and the number of households involved in rice production (72,400 households)^17^. The sample size obtained from the Eq. (1) is equal to 417.88 rice farmers to be surveyed. The choice of the surveyed villages was made in collaboration with the agents of the Territorial Agricultural Development Agencies (ATDA) based on rice production statistics, ease of access and the need for good country coverage. In total, 39 villages were selected for survey (Fig. 1).

### Surveys

Surveys were conducted from June 2019 to September 2019. In each selected village, at least 10 households were randomly selected using the transect method^18^ for individual interviews, for a total of 418 surveyed rice farmers. Due to ethnic diversity, local translators were recruited locally to facilitate discussions and exchanges with farmers. After a presentation of the research objectives to farmer, data were collected using a semi-structured questionnaire and related to: the socio-demographic and economic characteristics of each rice farmer respondent (age, sex, education, years of experience in rice production, training in rice production, household size, source of income, membership of a farmers’ association); rice production system; cultural practices (area sown, number of rice plots, type of cultivated rice varieties, bird control, frequency of fertilizer applications, type of labour, number of weeding, yield, number of ox-plough, straw management), and production constraints. At the level of each surveyed village, the altitude and geographical coordinates of two rice fields were collected using GPS (Global Positioning System).

### Data analysis

Data obtained during surveys were analysed by descriptive and multivariate statistics. Data on the socio-demographic profile of the surveyed rice farmers and the characteristics of the farms were subjected to Pearson *chi*-square tests and ANOVA using the IBM SPSS version 23.0 statistical software, in order to compare the different regions surveyed. The significance level was set at 0.05 and the means were separated by the Student Newman Keuls test^19^.

To classify the rice farming systems in the study area, analysis of survey data (Table 1) were performed in two steps: (1) a Factorial Analysis on Mixed Data (FAMD) was performed to produce an intermediate representation of the data; (2) then, a Hierarchical Cluster Analysis (AHC) was performed based on the "representative" factors of the FAMD. To identify the discriminant variables of the obtained clusters, a canonical discriminant analysis was performed. The identified rice farming systems were described and compared with each other using the finalfit package^20^. The map of rice farming systems was based on GPS surveys of rice fields in the surveyed villages. The map was created using QGIS 3.10.13 software (www.qgis.org).

**Table 1:** Description of variables used for rice farm characterization and as factors of adoption analysis of improved rice varieties.

Variables/characteristics	Codes	Definition	Measurement	Expected sign
**Dependant variable**				
Adoption of new rice variety	UIV	Adoption of new rice variety	1 if the farmer adopted improved rice variety, 0 otherwise	Nil
**Independent variables**				
*Socio-demographic factors*				
Proportion of male-headed households	MH	Gender of the household head	1 if respondent is male, 0 otherwise	+/−
Age of the household head	Age	Number of years from birth	Number	+/−
Education level of the household head	Education	Highest formal education level attained	1 if the farmers has a secondary education or higher education level, 0 if the farmer is illiterate or has a basic education	+
Household size	HS	Number of family members	Number	+/−
Experience in rice production	Experience	Number of years in rice farming	Number	+
Off-farm income	OFI	Other sources of farmer's income	1 if farmer has access to off farm income, 0 = Otherwise	−
*Farm resources factors*				
Land ownership	LO	The farmer owns the cultivated land	1 if the farmer owns land cultivated; 0 otherwise	+
Livestock ownership	LSO	Number of livestock own by the farmer	Number	+
Machinery ownership	MO	The farmer owns machinery (plow, tractors)	1 if the farmer owns any machinery, 0 otherwise	+
Total farm size	TFS	Hectares of farm plots cultivated	Hectares	+
Size of land under rice cultivation	LRS	Size of land under rice cultivation	Hectares	+
Total workforce	TW	Number of labour force used	Number	+
Family workforce	FW	Family workers	1 if the farmer used member of the household for farming, 0 otherwise	+
Hired farm labour	HFL	Farmer recruits persons outside the household for farming	1 if the farmer used other persons outside the household for farming, 0 otherwise	+
*Management factors*				
Crop diversification	RA	Growing of other crops in addition to rice	1 if there is risk averse, 0 otherwise	−
Training in rice farming	TRF	Farmer trained in rice production	1 if yes, 0 otherwise	
Membership of farmers association	MA	Member of farmers based organization	1 if yes, 0 otherwise	+
Rice as main crop	RMC	Rice is the main crop	1 if rice is the main crop for the household, 0 otherwise	+
Use of fertilizers	UF	Use of fertilizers by the farmers	1 if farmer use fertilizer, 0 otherwise	+
Use of pesticides	UP	Use of pesticides by the farmers	1 if farmer use pesticide, 0 otherwise	+
Animal traction	AT	Use of animal traction is used by the farmer	1 if animal traction is used by the farmer, 0 otherwise	+
Irrigation	Irrigation	Farming rice system is the irrigated system	1 if the farming rice is the irrigated system, 0 otherwise	+
Farmers output of rice	FOR	Quantity of rice harvested	Tonnes	+
Off-season rice	OSR	Production of rice in off-season	1 if farmer grows rice during off-season, 0 otherwise	+
*Institutional factors*				
Government extensions	GE	Farmer has contact with government extensions	1 if the farmer has contact with extension services, 0 otherwise	+
Non-governmental organizations	NGOS	Farmer has contact with an NGO	1 if the farmer has contact with NGOs, 0 otherwise	+
International institutes	InI	Farmer has contact with international institutes	1 if the farmer has contact with institutional institutes, 0 otherwise	+
**Geographical factors**	Region			
Dummy for the north region		The farmer’s region is the north	1 if the farmer’s region is the north, 0 otherwise	+/−
Dummy for the centre region		The farmer’s region is the centre	1 if the farmer’s region is the centre, 0 otherwise	+/−
Dummy for the south region		The farmer’s region is the south	1 if the farmer’s region is the south, 0 otherwise	+/−

From data collected a matrix of data composed of 418 rows representing the surveyed rice farmers and 28 columns representing the variables (quantitative and qualitative) was established. This data matrix was described from the cross sorting between the variable of interest (Adoption of improved rice) and each of the 27 other variables using the approach proposed by Xie^20^. This approach provides the means and the standard deviations of the continuous quantitative variables, a frequency table for the discontinuous and qualitative variables, followed by univariate tests on each variable. The effect of the different factors (variables) on the use of improved rice varieties was examined using a generalized linear fixed effect (all factors were fixed) model of binomial family. The model containing the twenty-seven (27) explanatory variables was first establish and the variance inflation factor (VIF) was examined for each variable in order to measure the collinearity. According to Hossain et al.^56^, if 0 < VIF < 5, there is no evidence of multi-collinearity. If 5 ≤ VIF ≤ 10, there is a moderate multi-collinearity, and finally if VIF > 10, there is high multi-collinearity between predictors. Due to the presence of the collinearity for many explanatory variables, a stepwise selection of variables was first made before adjusting the model to the data in order to avoid collinearity (correlations) between explanatory variables in the final model represented by the formula (Eq. 2):

2 $$ln\left[ {\frac{\pi \left( x \right)}{{1 - \pi \left( x \right)}}} \right] = \alpha_{0} + \alpha_{1} x_{1} + \alpha_{2} x_{2} + \cdots + \alpha_{15} x_{15} + \varepsilon$$

where $\pi \left(x\right)$ represents the probability of adopting the improved rice varieties by rice farmers knowing the vector of socio-cultural characteristics. The probability of adopting the improved rice varieties by rice farmers was expressed as a function of socio-cultural characteristics through the formula (Eq. 3):

3 $$\pi \left( x \right) = \frac{{\exp \left( {\alpha_{0} + \alpha_{1} x_{1} + \alpha_{2} x_{2} + \cdots + \alpha_{15} x_{15} } \right)}}{{1 + \exp \left( {\alpha_{0} + \alpha_{1} x_{1} + \alpha_{2} x_{2} + \cdots + \alpha_{15} x_{15} } \right)}}$$

The estimation of the coefficients ${\alpha }_{0},{\alpha }_{1},\dots ,{\alpha }_{15}$ was performed with the R software version 4.0.3 (http://CRAN.R-project.org/)^21^ using the maximum likelihood method. The description of the independent variables (${x}_{1},{\alpha }_{2},\dots ,{x}_{15})$ was presented in Table 1. The degree of susceptibility (likelihood) to use the improved varieties according to the selected factors was measured from the calculation of the odds ratios. Variables with a significant effect in the final model were identified from an overall test on the model.

The function *ktable* of the package knitr of R software version 4.0.3^20^ was used to describe the data matrix. The function *vif* of the package car^22^ was used to examine the multicollinearity of the explanatory variables. The selection of variables and the adjustment of the binomial regression to the data were carried out using the *glm* (generalized linear model) function of the package vgam^23^. The functions *tidy* of the package broom^24^, and *ggplot* of the package ggplot2^25^ were used to calculate and plot the odds ratios. The *drop1* function was used to identify variables with a significant effect in the model.

## Results

### Structural characteristics of rice farms

Men (74.6%) dominated rice production in the study area. The majority of the surveyed farmers (64.4%) had no formal education and average age of 43.9 years. Surveyed farmers in the southern region had significantly less experience in rice production than those in other regions (Table 2). The size of the surveyed households in northern and central Benin were significantly higher than those in southern. The surveyed farmers sowed an average area of 0.9 ha with the south having on average the largest plots sown by producers. Agriculture is the main source of income for the surveyed farmers and this in all the surveyed regions (Table 2). While the majority (71.8%) of surveyed farmers owned the land on which they grow rice, access to land remains a problem for a certain amount of them (22%), which cultivated rice on rented lands. In addition, there are few community-owned cultivation plots. In the Northern and Central regions of Benin, rice production was based on family labour force, while in the south the majority of farmers (53.2%) recruited workers. The majority of surveyed farmers (68.3%) were members of rice farmers’ cooperatives or associations. The lack of equipment for farmers in tractors, and ox-plough allowing the ploughing of fields is obvious in Republic of Benin but mostly in the southern and central regions. The great majority of surveyed farmers (64.6%) received at least one training in rice production or conservation and processing techniques or both (Table 3). However, there is a variation in trained farmers across the regions of Benin, as while the majority of surveyed farmers in the southern (85.5%) and central (71.7%) Benin have received training, it was the case only for 50.2% of them in the North. The structures involved in the training of rice farmers are mainly government agencies, NGOs, international institutions, farmer organizations and few agronomical companies (Table 3).

**Table 2:** Socio-demographics characteristics of surveyed farmers. (SE = Standard Error).

Characteristics	North (N = 227)	Centre (N = 53)	South (N = 138)	Study area (N = 418)	χ^2^-test	F-test
**Sex (%)**						
Male	74.9	69.8	76.2	74.6	0.809 ns	–
Female	25.1	30.2	23.9	25.4		
**Education (%)**						
Illiterate	69.2	62.3	57.2	64.4	12.482 ns	–
Primary	20.1	24.5	19.6	20.5		
Secondary	9.8	13.2	21	13.9		
University	0.9	–	2.2	1.2		
**Age (years)**						
Mean ± SE	43.6 ± 0.8	43.1 ± 1.1	47.6 ± 1.8	43.9 ± 0.6	–	2.648 ns
Range	[18–85]	[25–78]	[18–76]	[18–85]		
**Household size (%)**						
Mean ± SE	9.5 ± 0.4	7.7 ± 0.4	7.5 ± 0.3	8.6 ± 0.2	–	6.009**
Range	[1–34]	[2–15]	[1–24]	[1–34]		
**Experience (years)**						
Mean ± SE	15.1 ± 0.8	15.1 ± 1.9	11.5 ± 0.3	13.9 ± 0.8	–	3.479**
Range	[1–66]	[1–37]	[1–60]	[1–66]		
**Cultivated area (ha)**						
Mean ± SE	0.9 ± 0.0	1.2 ± 0.2	1.6 ± 0.3	0.9 ± 0.0	–	27.581***
Range	[0.05–16]	[0.25–5]	[0.25–8]	[0.05–16]		
**Access to land (%)**						
Owner	78.6	71.7	60.9	71.8	–	–
Rental	20	28.3	23.9	22.4		
Community	1.4	–	15.2	5.8		
**Total workforce (%)**						
Family	76.9	57.7	44.5	61.3	–	–
Paid worker	21.6	39.7	53.2	36.7		
Community	1.5	2.6	2.3	2		
**Sources of income (%)**						
Agriculture	93.3	84.9	97.9	93.7	–	–
Trade	5.1	7.5	0.7	4.1		
Transformation	–	5.7	0.7	0.9		
Hairdresser	0.4	1.9	–	0.5		
Welder	0.4	–	–	0.2		
Pension	–	–	0.7	0.2		
Carpenter	0.4	–	–	0.2		
Blacksmith	0.4	–	–	0.2		
**Membership of a rice farmers association (%)**						
Yes	58.2	52.8	88.4	68.3	–	–
No	41.8	47.2	11.6	31.7		
**Agricultural equipment (%)**						
Tractors	3.1	–	–	1.7	–	–
Plough	21.2	–	–	11.5		
Cattle	19.8	–	–	10.7		
None	55.9	100	100	76.1

**Table 3:** Structures involved in the training (production, conservation and processing techniques) of rice producers in the study area.

Region	Type of structure	Structures	Number of trained farmers
North (N = 114)	Government agencies	ATDA	74
		CPI	7
		ProCAD (PADA)	47
	NGOs	BORNE fonden	3
		GIZ (Pro-Agri, PROSOL)	21
	International institutions	AfricaRice	4
		CTB or ENABEL (PROFI)	4
		PNUD (PVM)	6
	Farmer organizations	URCPR-D	2
Centre (N = 38)	Government agencies	ATDA	22
		ProCAD (PADA)	2
	NGOs	Songhaï	1
		GIZ	18
		ONG '' Un monde''	1
		VECO-WA	1
	International institutions	AfricaRice	2
	Farmer organizations	UNIRIZ	3
		UCR	1
South (N = 118)	Government agencies	ATDA	91
		INRAB	3
		ProCAD (PADA)	9
		PAIA-VO	9
	NGOs	SNV	1
		GIZ	19
		ALDIPE	11
	International institutions	AfricaRice	2
		CTB or ENABEL	6
		IFDC	1
	Company	ESOP	4

ATDA: Territorial Agricultural Development Agencies, CPI: Investment Promotion Center, GIZ: German Technical Cooperation, ProAgri: Promotion of agriculture, ProCAD: Framework Support Program for Agricultural Diversification, PADA: Support Project for Agricultural Diversification, CTB or ENABEL: Belgian Technical Cooperation, PROFI: Agriculture support program, INRAB: National Institute for Agricultural Research of Benin, ESOP: Service Companies and Producers' Organizations, SNV: Dutch Development Organization, PAIA-VO: Agricultural infrastructure support project in the Valley of Ouémé, IFDC: International Center for Fertilizer Development. ALDIPE: Association for the Fight for Integrated Development and for the Protection of the Environment; UNIRIZ: Union of Hills Rice Producers; PVM: Millennium Villages Project, UCR: Communal rice farmers unions.

### Rice production

**Figure 2:**
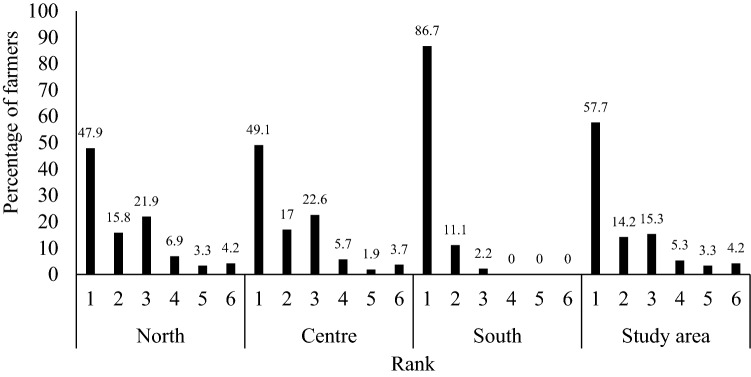
Rank occupied by rice production among surveyed farmers in function of rice-growing areas.

Rice is the main crop produced by the majority of the surveyed farmers (57.7%), and occupies the first place for the great majority of surveyed farmers (86.7%) in the southern Benin (Fig. 2). This trend is declining with 49.1% of surveyed farmers in the centre and 47.9% in the north having rice as main crop. Twenty-three other crops were listed as being produced by the surveyed farmers (Table 4). The yield of rice harvested in a season was estimated by the surveyed farmers to be around 2.3 tonnes/ha with a significantly (*p *< 0.000) higher production in the south (2.9 ± 0.1 tonnes/ha) than in the north (2.1 ± 0.2 tonnes/ha) and centre (1.6 ± 0.2 tonnes/ha) of Benin. The great majority (97%) of the surveyed farmers produced rice in lowland (Table 5), with only few surveyed farmers in the central (3.6%) and northern (4.6%) Benin producing upland rice. Rainfed rice production was the only type practiced by the surveyed farmers in central Benin. While, few farmers produced irrigated rice in the south (31.6%) and north (10.6%).

**Table 4:** Other plants cultivated by the surveyed farmers in the study area.

Crop	North (N = 227)	Centre (N = 53)	South (N = 138)	Study area (N = 418)
Maize	24.37	26.80	18.82	23.33
Soybean	8.73	17.01	5.62	10.45
Peanut	7.61	12.89	9.83	10.11
Yam	9.01	12.37	5.06	8.81
Cotton	12.25	4.64	5.90	7.60
Cassava	2.54	7.72	7.58	5.95
Cowpea	7.32	3.09	4.49	4.97
Sorghum	12.11	0.52	0.28	4.30
Millet	9.30	0.52	–	3.27
Pepper	1.69	4.12	3.37	3.06
Sesame	0.56		6.74	2.43
Tomato	–	0.52	6.46	2.33
Sweet potato	–	–	6.46	2.15
Oil palm	–	–	5.90	1.97
Cashew nut	0.56	4.64	–	1.73
Bambara groundnut	1.69	0.51	2.25	1.49
Kersting’s groundnut	0.15	1.03	3.09	1.42
Okra	–	1.55	2.25	1.27
Vegetable garden	–	0.52	3.09	1.20
Beans	0.28	1.03	1.12	0.81
Eggplant	–	0.52	1.69	0.74
Onion	1.13	–	–	0.38
Fonio	0.70	–	–	0.23

**Table 5:** Rice cropping systems and cultural practices used by rice farmers in the study area.

Practices	Modalities	Percentage of farmers			
		North (N = 227)	Centre (N = 53)	South (N = 138)	Study area (N = 418)
Culture zone	Lowland	95.4	96.4	100	97
	Upland	4.6	3.6	–	3
Rice production	Pluvial	89.4	100	68.4	83.2
	Irrigated	10.6	–	31.6	16.8
Type of irrigation	No	89.4	100	78.3	87.1
	Intermittent	10.6	–	10.1	9.1
	Continued	–	–	11.6	3.8
Type of produced rice	Local	39.9	–	–	21.7
	Improved	32.9	100	100	63.5
	Local and improved	27.2	–	–	14.8
Ploughing period	January–March	9.3	2	23.2	13.7
	April–June	82.3	74	53	70.3
	July–September	8.4	24	10.7	11.1
	October–December	–	–	13.1	4.9
Soil labour	Manual	56	96.4	95.1	73.7
	Plough	29.1	–	–	16
	Tractors	14.9	3.6	4.9	10.3
Sowing period	January–March	9.8	–	12.7	9.5
	April–June	75.1	17.6	33.6	54.4
	July–September	10.7	82.4	36.6	28
	October–December	4.4	–	17.1	8.1
Soil treatment before sowing	Yes	9.3	–	–	5.1
	No	90.7	100	100	94.9
Type of sowing	Sowing in pockets	79.7	80.9	24.5	61.4
	Nursery transplantation	16.8	19.1	75.5	36.8
	Broadcast sowing	3.5	–	–	1.8
Seedling spacing	10 × 10 cm	10.1	7.5	–	6.5
	15 × 15 cm	7.5	–	–	4.1
	20 × 20 cm	20.3	73.6	59.4	39.9
	25 × 25 cm	15.9	7.5	23.9	17.5
	30 × 30 cm	31.7	1.9	8.7	20.3
	40 × 30 cm	5.3	–	7.2	5.3
	Random	9.2	9.5	0.8	6.4
Cultural association	Yes	–	1.9	–	0.2
	No	100	98.1	100	99.8
Weed management	Manual	44.7	58.1	56.4	50.7
	Herbicide	55.3	41.9	43.6	49.3
Number of weeding	No weeding	60.4	1.9	3.6	33.3
	1	15.1	15.4	5.8	12.6
	2	22.7	46.2	44.2	32.9
	3	1.8	36.5	46.4	21.2
Soil fertility management	Chemical fertilizers	66.9	81.1	92.8	77.3
	Organic fertilizers	–	1.9	–	0.2
	No fertilizer	33.1	17	7.2	22.5
Insect pest management	Chemical pesticides	7.1	3.8	14.5	9.1
	No management	92.9	96.2	85.5	90.9
Months for bird scaring	January–March	0.4	–	19.2	9.1
	April–June	11	2.4	8.3	11.8
	July–September	80.2	92.7	59.2	66.3
	October–December	8.4	4.9	13.3	12.8
Harvest period	January–April	3.9	–	20.2	9.2
	May–August	19.8	1.9	26.8	20.3
	September–December	76.3	98.1	53	70.6
Post-harvest straw management	Arrange in a pile in the fields	76.7	83.3	90.6	82.1
	Remove in the fields	4.4	16.7	9.4	7.6
	Burn	2.6	–	–	1.4
	Compost	16.3	–	–	8.9

### Rice cultural practices

The interval from April to June is the main ploughing period in the study area. However, the practice of irrigated rice production allows farmers in southern Benin to produce rice in the off-season. The majority of farmers manually performed land preparation, with some use of ox-plough for ploughing in the north of Benin. A great majority of the surveyed farmers hired tractors to plough the rice fields. Before sowing, only a few farmers in the North (9.3%) treated the soils with herbicides. To dig seedling holes farmers used diverse craft tools. Semi in pockets was the main method of sowing practiced by the surveyed farmers in northern and central Benin, while the majority of surveyed farmers in the south (75.5%) of Benin set up nurseries and then transplant the young plants. The sowing distances practiced varied from one prospected region to another. The majority of farmers in southern and central Benin use a spacing of 20 × 20 cm between plants, while in the north the spacing between plants varied considerably, or even being random (Table 5). All the surveyed farmers in the south and centre Benin cultivated only improved varieties. However, in the north, farmers produced both local (39.9%) and improved rice varieties (32.9%).

Rice is cultivated in monoculture in almost all production areas, only one surveyed farmer from central Benin cultivating rice in association with yam. Weed management after sowing is mainly performed using herbicides in northern Benin (55.3% of farmers), with manual weeds removal in the rice fields by the majority of farmers in the southern (56.4%) and central (58.1%) regions of Benin. During the rice vegetative stage, most of the surveyed farmers (60.4%) in the north Benin do not clean weeds, while farmers in southern and central Benin do 2 to 3 weeding. Chemical fertilizers are used for soil fertilization by most of the surveyed farmers. The great majority (90.9%) of surveyed farmers do not use any method of pest management in the rice fields. The hunting of pest birds usually takes place between July and September. Harvests are mainly done between September and December and that across all regions. After the harvest, the majority of the rice farmers (82.1%) leaved the rice straws in the fields, only a few surveyed farmers in northern Benin (16.3%) transforming straw into compost.

### Characterisation of rice production systems

Taking into account the three rice-cropping systems (rainfed lowland, rainfed upland and irrigated lowland) registered in the study area (Fig. 1), we noted a variation in cultivation practices from one system to another (Table 5). Indeed, farmers practicing rainfed and irrigated lowland rice farming tend to cultivate improved varieties compared to those practicing rainfed upland rice productions. Moreover, farmers practicing irrigated lowland rice production used more sowing than transplantation. Little difference was observed between the proportion of farmers practicing the different rice production systems in terms of ploughing, soil fertility management or pest management (Table 6).

**Table 6:** Agricultural practices of farmers in function of rice production systems.

Practices	Modalities	Rainfed lowland (N = 381)	Rainfed upland (N = 14)	Irrigated lowland (N = 73)
Soil labour	Manual	290	13	48
	Plough	66	1	23
	Tractors	39	3	3
Type of produced rice	Local	79	11	16
	Improved	302	3	57
Type of sowing	Sowing in pockets	260	13	8
	Nursery transplantation	130	2	71
	Broadcast sowing	8	–	–
Weed management	Manual	273	7	57
	Herbicide	270	11	51
Soil fertility management	Chemical fertilizers	290	4	67
	Organic fertilizers	1	–	–
	No fertilizer	90	10	6
Insect pest management	Chemical pesticides	28	–	21
	No management	353	14	52

**Figure 3:**
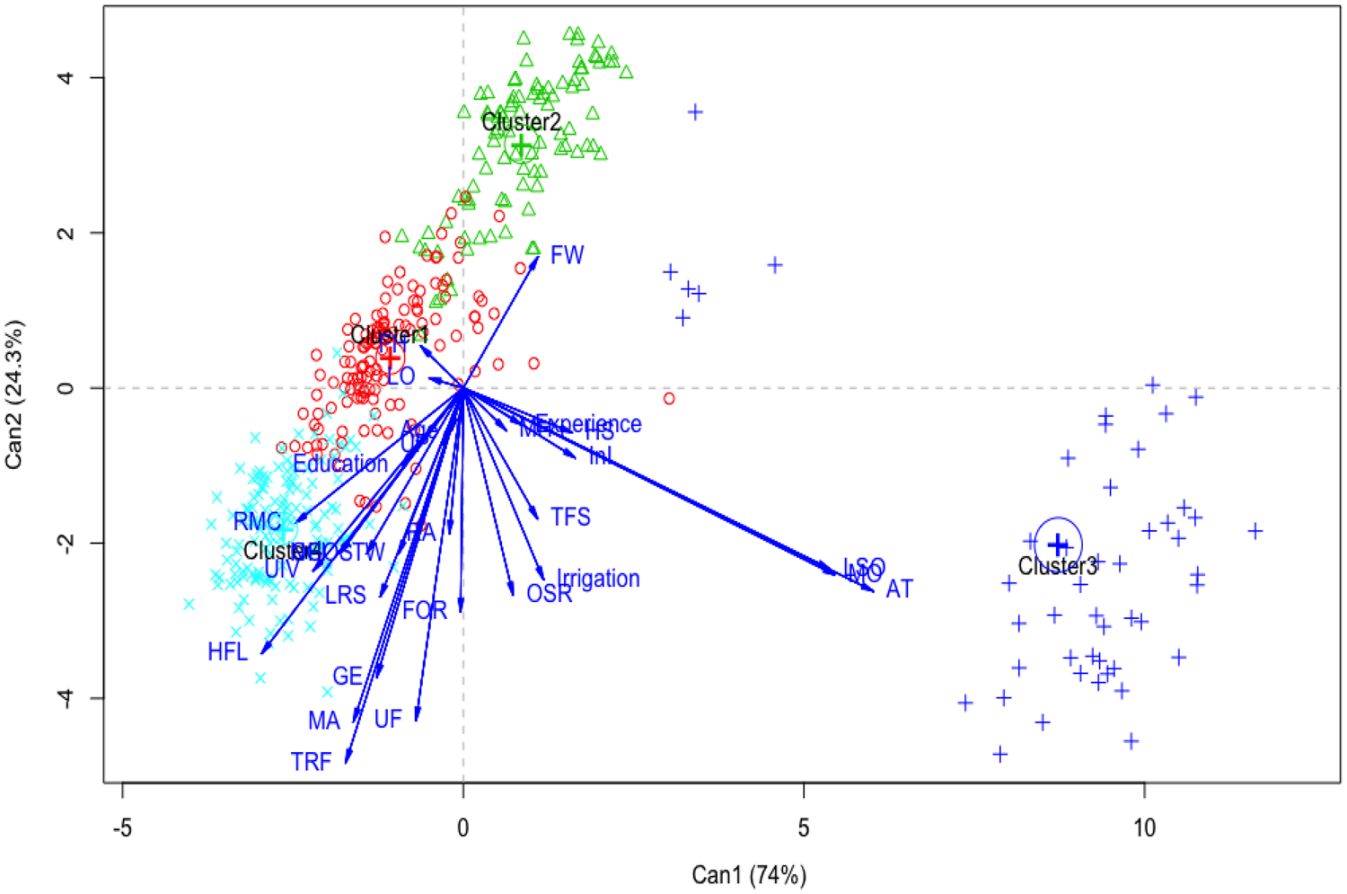
Position of rice farming systems on the first and second factors (Dimension 1 and 2) derived from canonical discriminant analysis.

The hierarchical cluster analysis showed four significant clusters of rice farming systems. Canonical discriminant analysis revealed that the first two canonical axes were globally significant (*p* < 0.05) with 74% for the first axis and 24.3% for the second (Fig. 3). These two canonical axes suffice to identify the variables that distinguish the rice farmers’ farming systems. The animal traction possession (AT), livestock ownership (LSO) and machinery ownership (MO) were significantly correlated with the first canonical axis (Table 7). The second canonical axis were significantly correlated with the variables government extensions (GE), hired farm labor (HFL), membership association (MA), training rice farming (TRF) and use fertilizer (UF) (Table 7). These eight (8) variables are the most discriminating of the four rice-farming systems. Clusters 1 and 4 are opposed to cluster 3 on the first axes. Rice production system 3 brings together predominantly with rice farmers who own animal traction (AT) and machines (MO) but also a high number of livestock (LSO) unlike those in groups 1 and 4. Rice farming systems 1, 3 and 4 consist predominantly of rice farmers belonging to the government extensions (GE) and association member (MA), having hired agricultural labor (HFL) and training on rice cultivation (TRF) but also use fertilizer (UF) unlike those in group 2 (Fig. 3). According to Table 8, the rice farming systems that emerge are:

- Semi-intensive rice farming system practiced by 133 (31.8%) surveyed farmers, spread across all prospected rice-growing areas and characterized by average sown area, use of fertilizer, pesticides and improved rice varieties, but little use of irrigation systems and hired labour (cluster 1).
- Subsistence or traditional rice farming system, in lowlands, on small farm size mainly practiced by 95 (22.7%) surveyed farmers from the north Benin using only a family workforce. In this system, the surveyed farmers were not organize in associations and don’t use irrigation systems and improved varieties, which underlines their low yield (cluster 2).
- Integrated rice–livestock farming system based on the use of animal traction and mechanical equipment (cluster 3). Only practiced by 49 (11.8%) surveyed farmers from the north.
- Intensive rice farming system practiced by 141 (33.7%) trained farmers on rice production techniques from the south Benin using chemical inputs and improved varieties (cluster 4). For these farmers, rice is the main crop, grown on big farm size and employing a high number of hired labor.

**Table 7:** Correlation between canonical axes and variables.

Variables	Axe 1	Axe 2	Axe 3
Age	0.026	0.084	0.186
Animal traction	**− 0.897**	0.390	**− **0.004
Education	0.135	0.149	**− **0.062
Experience	**− **0.128	0.071	0.201
Female-headed households	0.094	**− **0.081	0.122
Farmers output of rice	0.006	0.431	**− **0.257
Family workforce	**− **0.163	**− **0.252	0.063
Government extensions	0.189	**0.557**	0.121
Hired farm labour	0.442	**0.510**	**− **0.061
Household size	**− **0.238	0.086	0.175
International institutes	**− **0.246	0.134	0.180
Irrigation	**− **0.176	0.369	**− **0.238
Land ownership	0.075	**− **0.019	0.093
Size of land under rice cultivation	0.182	0.402	**− **0.284
Livestock ownership	**− 0.802**	0.348	0.047
Membership of farmers association	0.240	**0.641**	0.085
Male-headed households	**− **0.094	0.081	**− **0.122
Machinery ownership	**− 0.813**	0.359	0.159
Non-governmental organizations	0.211	0.318	**− **0.047
Off-farm income	0.270	0.318	**− **0.081
Off-season rice	**− **0.109	0.399	**− **0.309
Crop diversification	0.031	0.281	0.237
Rice as main crop	0.368	0.259	**− **0.337
Total farm size	**− **0.163	0.252	0.142
Training in rice farming	0.257	**0.720**	0.303
Total workforce	0.142	0.319	**− **0.239
Use of fertilizers	0.104	**0.639**	0.403
Adoption of new rice variety	0.329	0.352	0.152
Use of pesticides	0.045	0.109	0.054

**Table 8:** Comparison of qualitative and quantitative variables between rice farming systems.

		Clusters				*p*
		C1	C2	C3	C4	
**Qualitative variables**						
Education	Illiterate	89 (66.9)	68 (71.6)	35 (71.4)	77 (55.4)	0.018
	Primary	25 (18.8)	21 (22.1)	10 (20.4)	28 (20.1)	
	Secondary	16 (12.0)	6 (6.3)	4 (8.2)	32 (23.0)	
	University	3 (2.3)	0 (0.0)	0 (0.0)	2 (1.4)	
FH	No	92 (69.2)	69 (72.6)	43 (87.8)	106 (76.3)	0.074
	Yes	41 (30.8)	26 (27.4)	6 (12.2)	33 (23.7)	
FW	No	23 (17.3)	4 (4.2)	6 (12.2)	45 (32.4)	< 0.001
	Yes	110 (82.7)	91 (95.8)	43 (87.8)	94 (67.6)	
GE	No	63 (47.4)	89 (93.7)	23 (46.9)	30 (21.6)	< 0.001
	Yes	70 (52.6)	6 (6.3)	26 (53.1)	109 (78.4)	
HFL	No	68 (51.1)	87 (91.6)	40 (81.6)	18 (12.9)	< 0.001
	Yes	65 (48.9)	8 (8.4)	9 (18.4)	121 (87.1)	
InI	No	127 (95.5)	95 (100.0)	40 (81.6)	137 (98.6)	< 0.001
	Yes	6 (4.5)	0 (0.0)	9 (18.4)	2 (1.4)	
Irrigation	No	128 (96.2)	95 (100.0)	30 (61.2)	109 (78.4)	< 0.001
	Yes	5 (3.8)	0 (0.0)	19 (38.8)	30 (21.6)	
LO	No	27 (20.3)	24 (25.3)	16 (32.7)	33 (23.7)	0.377
	Yes	106 (79.7)	71 (74.7)	33 (67.3)	106 (76.3)	
AT	No	133 (100.0)	95 (100.0)	6 (12.2)	139 (100.0)	< 0.001
	Yes	0 (0.0)	0 (0.0)	43 (87.8)	0 (0.0)	
MA	No	48 (36.1)	81 (85.3)	18 (36.7)	6 (4.3)	< 0.001
	Yes	85 (63.9)	14 (14.7)	31 (63.3)	133 (95.7)	
MH	No	41 (30.8)	26 (27.4)	6 (12.2)	33 (23.7)	0.074
	Yes	92 (69.2)	69 (72.6)	43 (87.8)	106 (76.3)	
MO	No	125 (94.0)	95 (100.0)	5 (10.2)	138 (99.3)	< 0.001
	Yes	8 (6.0)	0 (0.0)	44 (89.8)	1 (0.7)	
NGOS	No	109 (82.0)	95 (100.0)	44 (89.8)	89 (64.0)	< 0.001
	Yes	24 (18.0)	0 (0.0)	5 (10.2)	50 (36.0)	
OFI	No	103 (77.4)	92 (96.8)	45 (91.8)	77 (55.4)	< 0.001
	Yes	30 (22.6)	3 (3.2)	4 (8.2)	62 (44.6)	
OSR	No	123 (92.5)	92 (96.8)	27 (55.1)	92 (66.2)	< 0.001
	Yes	10 (7.5)	3 (3.2)	22 (44.9)	47 (33.8)	
RA	No	11 (8.3)	27 (28.4)	3 (6.1)	9 (6.5)	< 0.001
	Yes	122 (91.7)	68 (71.6)	46 (93.9)	130 (93.5)	
RMC	No	65 (48.9)	54 (56.8)	36 (73.5)	16 (11.5)	< 0.001
	Yes	68 (51.1)	41 (43.2)	13 (26.5)	123 (88.5)	
TRF	No	36 (27.1)	87 (91.6)	16 (32.7)	3 (2.2)	< 0.001
	Yes	97 (72.9)	8 (8.4)	33 (67.3)	136 (97.8)	
UF	No	19 (14.3)	64 (67.4)	4 (8.2)	4 (2.9)	< 0.001
	Yes	114 (85.7)	31 (32.6)	45 (91.8)	135 (97.1)	
UIV	No	19 (14.3)	43 (45.3)	19 (38.8)	1 (0.7)	< 0.001
	Yes	114 (85.7)	52 (54.7)	30 (61.2)	138 (99.3)	
UP	No	41 (30.8)	39 (41.1)	16 (32.7)	38 (27.3)	0.169
	Yes	92 (69.2)	56 (58.9)	33 (67.3)	101 (72.7)	
**Quantitative variables**						
Age	Mean (SD)	45.3 (12.3)	41.3 (13.0)	44.0 (12.7)	44.1 (12.7)	0.127
HS	Mean (SD)	8.7 (5.3)	7.9 (3.7)	11.7 (5.3)	7.8 (4.2)	< 0.001
LSO	0 (%)	130 (97.7)	95 (100.0)	8 (16.3)	139 (100.0)	< 0.001
	2 (%)	3 (2.3)	0 (0.0)	27 (55.1)	0 (0.0)	
	3 (%)	0 (0.0)	0 (0.0)	1 (2.0)	0 (0.0)	
	4 (%)	0 (0.0)	0 (0.0)	13 (26.5)	0 (0.0)	
TFS	Mean (SD)	4.3 (5.7)	2.2 (1.8)	7.6 (8.6)	4.5 (4.6)	< 0.001
LRS	Mean (SD)	0.9 (0.7)	0.5 (0.3)	1.1 (0.8)	1.9 (1.9)	< 0.001
TW	Mean (SD)	6.3 (4.7)	3.9 (2.3)	7.6 (9.2)	13.2 (16.6)	< 0.001
FOR	Mean (SD)	1.7 (1.7)	1.1 (1.2)	3.2 (2.5)	3.3 (2.7)	< 0.001
Experience	Mean (SD)	14.8 (11.4)	12.3 (11.3)	18.0 (12.6)	12.8 (8.0)	0.008

### Constraints of rice production

Fifteen constraints related to rice production were identified across the study area (Table 9). All of listed constraints were found in southern Benin, but only 13 and 9 of them were identified respectively in northern and central Benin, respectively. Lack of farm machinery and agricultural credit were the main constraints in rice production across all regions. The maintenance of fields and the lack of workers are significant constraints in the south and centre regions of Benin. As for the north, the increase in the price of inputs was considerably slowing rice production. Poor water management, drought, and bird attacks on rice fields were constraints also identified in all surveyed regions. The lack of a sales market, insect pest attacks, lack of usable land and soil infertility were constraints found only in the north and south of Benin. While, the lack of irrigation system was identified as constraint only in central and southern Benin.

**Table 9:** Constraints of rice production in the study area.

Constraints	North	Centre	South	Study area
Lack of farm machinery	20	23.8	20.1	20.6
Lack of agricultural credit	22.4	19.8	11.1	18.2
Field maintenance	5.1	17.8	16.8	10.8
Increase of input prices	21.1	3	9	14.4
Lack of manpower	6.1	12.9	11.9	9
Poor water management	4	8.9	2.9	4.3
Bird attacks	2.9	8.9	6.1	4.9
No sales market	6.1	–	7	5.6
Pest attacks	4	–	2.9	3.1
Lack of rice cooperative	–	–	7.4	2.5
Poor seed quality	2.6	–	2.8	2.4
Lack of irrigation system	–	3.9	0.8	0.8
Drought	1.9	1	0.4	1.2
Lack of exploitable land	1.9	–	0.4	1.1
Soil infertility	1.9	–	0.4	1.1

### Factors affected the use of improved rice varieties

**Figure 4:**
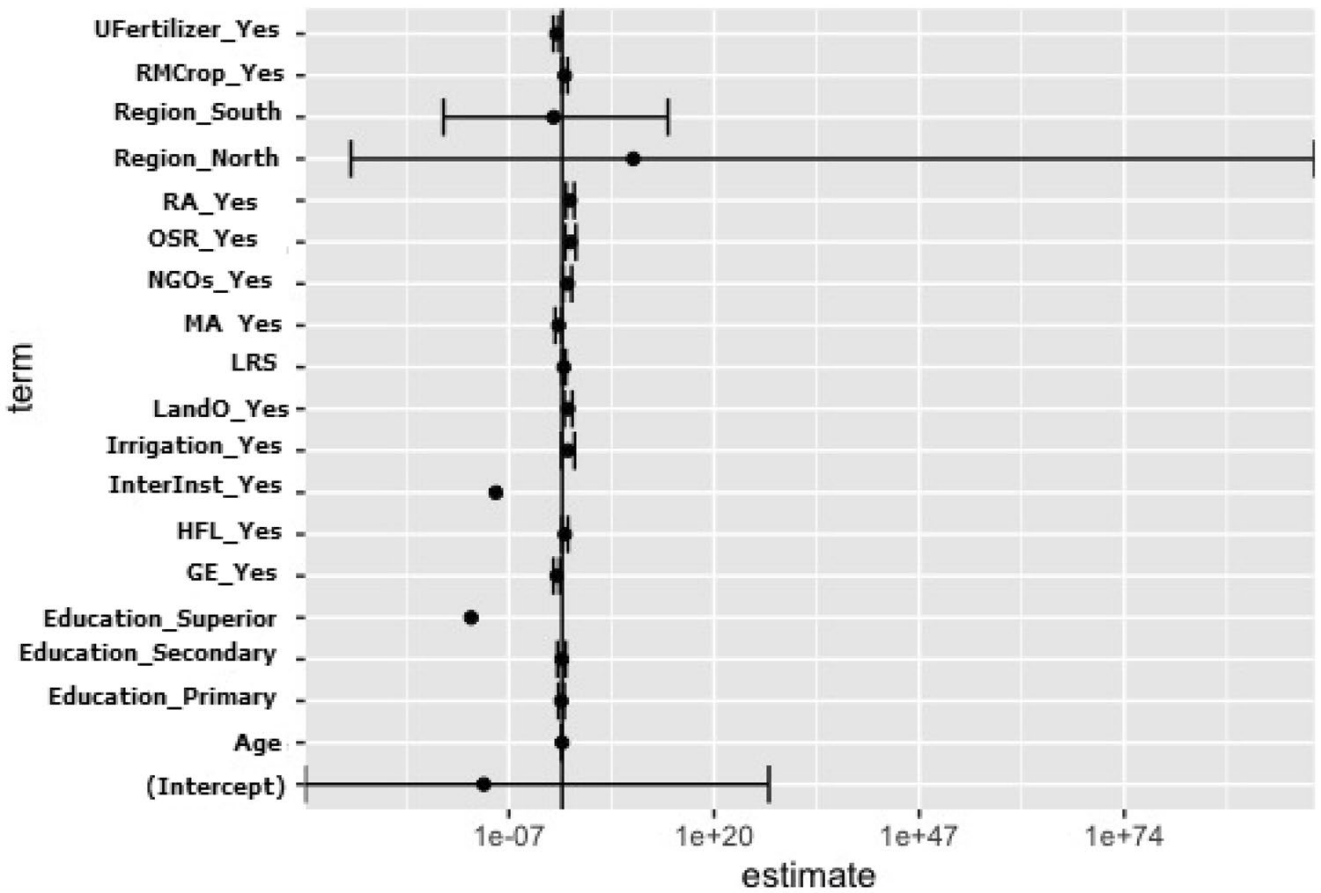
Graphical representation of odds ratios. Ufertilizer: use of fertilizer, RMcrop: rice as main crop, RA: crop diversification, OSR: off-season rice, MA: Membership of association, LRS: land size under rice cultivation, LandO: land ownership, INterInst: contact with international institution, HFL: hired farm labour, GE: contact with government extensions.

Rice farmers using at least one variety of improved rice were significantly (*p* < 0.05) older, but belonged to households of small size, compared to those who did not use any improved varieties at all (Table 10). Use or not of at least one improved variety of rice by farmers was significantly (*p* < 0.05) related to the off farm income, the hired farm labour, the training rice farming, the membership association, the rice production as main crop, the use of fertilizer, the contact with government extensions, NGOs, and international institutes, and the farmers’ region (Table 10). When assessing the factors significantly influencing the adoption of improved rice varieties, the stepwise selection allowed us to select fifteen factors (Akaike information criterion = 245.16 for the saturated model and 231.09 after the selection of the fifteen factors). The detailed results of the binomial regression model (Table 11) showed that multiple factors affected the adoption, or non-adoption, of improved rice varieties. Rice farmers in contact with NGOs were more likely to adopt at least one improved rice varieties. In contrast, membership of farmers association and contact with government extensions was negatively related to the adoption of improved rice. Rice farmers with land ownership are more likely to adopt improved rice, and the crop diversification and the use off-season rice were also positively related to the adoption of improved rice varieties. At the opposite rice farmers, which use less fertilizer are unlikely to adopt improved rice. According to the Fig. 4, rice farmers cultivating a diversity of crops or producing off-season rice or in contact with NGOs or with land ownership were respectively 10.6, 12, 4.93 and 5.83 times more likely to adopt improved rice than those presenting opposite profile. The result of the analysis relating to the identification of significant variables in the model shows that the deletion of the factors: age, hired farm labour, rice main crop and irrigation does not significantly modify the model, indicating the absence of effect of these variables (Table 12).

**Table 10:** Summary statistics for adopters and non-adopters of improved rice varieties.

Variables		Adopters (N = 335)		Non-adopters (N = 82)		Probability
Age	Mean (SD)	44.7	(12.3)	40.9	(13.0)	**0.014**
Education	Illiterate	213	(63.6)	56	(68.3)	0.417
	Primary	67	(20.0)	18	(22.0)	
	Secondary	50	(14.9)	8	(9.8)	
	University	5	(1.5)	0	(0.0)	
Household size	Mean (SD)	8.3	(4.6)	9.7	(5.1)	**0.015**
Experience	Mean (SD)	13.7	(10.0)	15.0	(12.8)	0.339
Off-farm income	No	242	(72.2)	76	(92.7)	**< 0.001**
	Yes	93	(27.8)	6	(7.3)	
Land ownership	No	85	(25.4)	15	(18.3)	0.229
	Yes	250	(74.6)	67	(81.7)	
Livestock ownership	0	304	(90.7)	69	(84.1)	0.240
	2	20	(6.0)	10	(12.2)	
	3	1	(0.3)	0	(0.0)	
	4	10	(3.0)	3	(3.7)	
Machinery ownership	No	298	(89.0)	66	(80.5)	0.060
	Yes	37	(11.0)	16	(19.5)	
Total farm size	Mean (SD)	4.3	(5.4)	4.5	(5.2)	0.757
Land size under rice cultivation	Mean (SD)	1.2	(1.4)	1.0	(1.0)	0.099
Total workforce	Mean (SD)	8.5	(11.6)	6.8	(8.3)	0.235
Family workforce	No	69	(20.6)	10	(12.2)	0.113
	Yes	266	(79.4)	72	(87.8)	
Hired farm labour	No	156	(46.6)	58	(70.7)	**< 0.001**
	Yes	179	(53.4)	24	(29.3)	
Crop diversification	No	43	(12.8)	7	(8.5)	0.376
	Yes	292	(87.2)	75	(91.5)	
Training in rice farming	No	85	(25.4)	58	(70.7)	**< 0.001**
	Yes	250	(74.6)	24	(29.3)	
Membership of farmers association	No	99	(29.6)	55	(67.1)	**< 0.001**
	Yes	236	(70.4)	27	(32.9)	
Rice as main crop	No	125	(37.3)	47	(57.3)	**0.002**
	Yes	210	(62.7)	35	(42.7)	
Use of fertilizer	No	56	(16.7)	36	(43.9)	**< 0.001**
	Yes	279	(83.3)	46	(56.1)	
Use of pesticides	No	107	(31.9)	28	(34.1)	0.802
	Yes	228	(68.1)	54	(65.9)	
Animal traction	No	305	(91.0)	70	(85.4)	0.185
	Yes	30	(9.0)	12	(14.6)	
Irrigation	No	297	(88.7)	67	(81.7)	0.131
	Yes	38	(11.3)	15	(18.3)	
Farmers output of rice	Mean (SD)	2.4	(2.3)	2.0	(2.9)	0.211
Off-season rice	No	275	(82.1)	61	(74.4)	0.154
	Yes	60	(17.9)	21	(25.6)	
Government extensions	No	142	(42.4)	64	(78.0)	**< 0.001**
	Yes	193	(57.6)	18	(22.0)	
NGOs	No	264	(78.8)	74	(90.2)	**0.027**
	Yes	71	(21.2)	8	(9.8)	
International institutes	No	318	(94.9)	82	(100.0)	**0.077**
	Yes	17	(5.1)	0	(0.0)	
Regions	Centre	42	(12.5)	0	(0.0)	**< 0.001**
	North	154	(46.0)	82	(100.0)	
	South	139	(41.5)	0	(0.0)	

Probability values that are significant at 0.05 level are in bold.

**Table 11:** Factors affecting adoption of improved rice varieties in the study area.

Variables	Estimate	Std. Error	z value	Pr (> z)
Intercept	− 23.744	2334.064	− 0.010	0.992
Age	− 0.026	0.015	− 1.682	0.093
Education-Primary	− 0.277	0.439	− 0.631	0.528
Education-Secondary	− 0.131	0.618	− 0.212	0.832
Education-University	− 27.601	5110.371	− 0.005	0.996
**Land ownership—Yes**	**1.764**	**0.625**	**2.821**	0.005**
Land rice size	0.500	0.258	1.935	0.053
Hired farm labour—Yes	0.819	0.461	1.776	0.076
**Crop diversification—Yes**	**2.356**	**0.634**	**3.719**	0.000***
**Membership association—Yes**	− **1.075**	**0.510**	− **2.109**	0.035*
Rice as main crop—Yes	0.799	0.425	1.879	0.060
**Use of fertilizer—Yes**	− **1.724**	**0.460**	− **3.749**	0.000***
Irrigation—Yes	1.761	1.013	1.738	0.082
**Off-season rice—Yes**	**2.482**	**0.807**	**3.077**	0.002**
**Government extensions—Yes**	− **1.593**	**0.509**	− **3.131**	0.002**
**NGOs—Yes**	**1.594**	**0.670**	**2.379**	0.017*
International institutes—Yes	− 20.085	3549.896	− 0.006	0.995
Region—North	21.578	2334.064	0.009	0.993
Region—South	− 2.574	2577.223	− 0.001	0.999

Std.Error: Standard Error; Pr (> z): Probability. Probability values that are significant at 0.05 level are in bold.

**Table 12:** Marginal effect analysis on determinants of adoption of improved rice varieties.

Variables	Df	Deviance	AIC	LRT	Pr(> Chi)
< None >		193.09	231.09		
**Age**	**1**	**196.04**	**232.04**	**2.954**	**0.086**
Education	3	201.70	233.70	8.611	0.035*
Land ownership	1	202.69	238.69	9.603	0.002**
Land size under rice cultivation	1	197.58	233.58	4.495	0.034*
Hired farm labour	**1**	**196.28**	**232.28**	**3.195**	**0.074**
Crop diversification	1	209.06	245.06	15.972	6.43e−05***
Membership association	1	197.57	233.57	4.478	0.034*
Rice as main crop	**1**	**196.74**	**232.74**	**3.647**	**0.057**
Use of fertilizer	1	208.18	244.18	15.094	0.000***
**Irrigation**	**1**	**196.16**	**232.16**	**3.067**	**0.080**
Off-season rice	1	202.76	238.77	9.677	0.002**
Government extensions	1	203.76	239.76	10.673	0.001**
NGOs	1	198.54	234.54	5.450	0.020*
International institutes	1	204.86	240.86	11.775	0.001***
Region	2	308.75	342.75	115.658	<2.2e−16***

AIC: Akaike Information Criterion; LRT: Likelihood Ratio Tests; Pr(> Chi): Probability. Probability values.

### Ethical approval and informed consent

The research protocol was approved by the ethic committee of the National University of Sciences, Technologies, Engineering and Mathematics (UNSTIM). Interviews were carried out in accordance with the guidelines of the Declaration of Helsinki. Written informed consent was obtained from all participants prior to the interviews.

### Consent to participate

Informed consent was obtained from all participants prior to the interviews.

## Discussion

Our results showed that men dominate rice production in the study area. Indeed, Kinkingninhoun-Mêdagbé et al.^26^ observed that there is great discrimination against women rice farmers with regard to access to land in the Republic of Benin. Beninese women are however more involved in latter steps, *i.e.* the processing and marketing of rice^27^. The low experience of farmers of southern Benin in rice production, compared to those of other regions, could be explained by a more recent introduction of rice production in this region^9^. Indeed, Vido^28^ noted that the production of African rice (*O. glaberrima*) takes place in central and northern Benin, long before the colonial era. The fact that the majority of surveyed farmers own their rice land positively influences rice production in the study area. Indeed, owning their rice fields allows rice farmers to make long-term investments (such as investment in irrigation technologies), leading to an increase in rice production^29,30^.

Our study showed that rice is a very important crop for the majority of surveyed farmers, particularly for those of southern region where it is the main crop produced. As perceived by the surveyed farmers and corroborated by FAO statistics, rice production in the Republic of Benin has increased rapidly between 2015 and 2019 from 204,310 to 406,000 tonnes^1^. However, the number of tonnes of rice produced per hectare declared by the surveyed farmers in northern Benin is significantly lower comparatively to those of southern Benin. This could be explained by the use of fertilizer by the majority of surveyed farmers in the southern region and the high number of weeding practised by these farmers. Indeed, soil fertility and weed management are the main cause of rice yield gaps^31^. Moreover, the great majority of surveyed farmers in south region were trained by various structures on rice production, which has been shown to have significantly positive impacts on rice yield^32^. In addition, it is in the southern region that we surveyed the most farmers practicing irrigated rice cultivation, increasing again the productivity^33^. Therefore, to boost rice production in Republic of Benin it is important that structures involved in rice farmers training (government agencies, NGOs, international institutions, farmer organizations and agronomical companies) train them to the irrigated rice system practices.

Only three rice cropping system were practiced in the study area comparing to the neighbouring country, Nigeria, where five rice production systems have been registered^34^. However, the dominance of lowland rainfed rice production was also found in many others West Africa countries^29^, while this system of rice production is highly dependent of the duration of raining season, frequently disturbed in Republic of Benin due to the climate change^35^. It is known that, the establishment of irrigation systems is a major pre-requirement to attain rice green revolution^8^. Therefore, government actions such as subsidies allowing the acquisition of equipment for new irrigation and water saving technologies should be strengthened.

The great majority of surveyed farmers practiced rice monoculture. While, it is known that the rice monoculture does not allow maximum use of the potential of lowland soil resources^36^, and leads over the years to a decrease in rice yield^37^. Indeed, intercropping rice and pigeon pea or maize significantly increases grain yield of rice, reduce nematode infestation of rice and weed biomass compared to rice grown in monoculture^38^. Therefore, it is important that agents of the Territorial Agricultural Development Agencies (ATDA) of each rice-growing areas, and scientist train Beninese rice farmers on rice intercropping practices and convince them on the economic returns that their choice can generate.

Our results showed that traditional rice farming system is widely practiced in northern Benin, and therefore underline the low yields observed in the region. It is therefore important to intensify the action of extension services in this region through the training of farmers on modern production techniques (irrigation, use of inputs, etc.). Linking rice farmers through farmers' organizations or cooperatives is necessary to strengthen their access to information on these modern production technologies, and credit facilities from local financial institutions. Indeed, Van Campenhout^39^ showed that rice farmers associations play an important role in the dissemination of agricultural information and the adoption of modern agronomic practices. The integrated rice–livestock farming system practiced by some surveyed farmers in the north Benin must be encouraged because this integrated farming system is known to improve household income, food security, and environmental sustainability^40^. The strengthening of semi-intensive and intensive rice-growing systems can be done through the provision of agricultural machinery to farmers' organizations or cooperatives to facilitate the plowing of fields.

Similarly to Angola rice production system^7^, a weak mechanization of rice production was observed as the main constraints in all the study area. Indeed, the adoption of agricultural machinery allows an increase in yield and incomes^41^. This lack of farm machinery combined with the poor management of insect pests and diseases contributes and other factors to low rice productivity in Republic of Benin. Nonvide et al.^57^ in the municipality of Malanville (northern Benin) also mentioned the importance of agricultural credit as constraints of rice production. Therefore, it is important to set up a formal credit system for rice farmers allowing them to face the various costs related to rice production, such as equipment in agricultural machinery, payment of labour used, purchase farm inputs, etc. Agricultural credit was found as the most important factor to boost rice production in several countries such as Ethiopia^42^, and Pakistan^43^.

The use of improved rice varieties is a reality in the Republic of Benin with the majority of surveyed farmers cultivating at least one improved variety. Only improved rice varieties are cultivated by the surveyed farmers in southern and central Benin, suggesting a market-oriented rice production. Indeed, the quality of local rice varieties was not very appreciated by Beninese consumers who prefer long-grain flavoured white rice^44,45^. Therefore, the improved variety IR841 meeting consumer requirements is now widely cultivated by Beninese farmers^9,46^. The coexistence of improved rice varieties and local landraces in northern Benin underlines the strong cultural anchoring of local landraces^9^. Naseem et al.^44^ noted the low consumption of improved rice in the northwest Benin due to the subsistence living conditions of farmers and inaccessibility of villages due to poor roads.

Older surveyed farmers adopted significantly improved varieties than younger. This could be explained by the fact that the longevity of producers exposes them to more agricultural innovations and therefore to their adoption^47^. Similarly, the surveyed households having few people adopted more improved rice varieties. Indeed, according to Bruce et al.^48^, the pressure of the financial burdens associated with large families does not allow them to invest in new technologies such as improved rice varieties. The surveyed farmers using hired farm labour adopted more improved rice varieties probably because improved rice is cultivated on large areas and is labour-intensive than growing local rice. The surveyed farmers who had received training in rice production or who were members of a farmers' association adopted the improved rice varieties more than those with the opposite profile. This is not surprising because it is known that regular contact with extension organizations (government extensions, NGOS, and international institutes), and participation to farmers’ association meetings allow farmers to have information about new technologies such as improved rice varieties and promote their adoption^5,47,49^. The surveyed farmers with rice as main crop and off farm income adopted more improved varieties. As suggested by Hagos and Zemedu^50^, alternative income sources allows farmers to acquire the inputs such as seed and fertilizers and hired additional labour necessary for production of improved rice varieties. Indeed, off-farm incomes are an important strategy helping to overcome the financial constraints faced by smallholder farmers^51^.

Our results show that farmers who practice off-season rice are 12 times more likely to adopt improved varieties. In fact, the shorter growth duration of improved rice varieties allows farmers to produce a second rice crop^52^. Likewise, the land ownership positively influences and multiplies by 5.83 the adoption of improved rice varieties by Beninese farmers. Indeed, Bruce et al*.*^48^ reported that farmers with secure land tenure adopt new technologies because they have the capacity to face losses if the technologies fail. Similarly to Indian rice farmers^53^ the crop diversification influenced positively the adoption of improved rice varieties. The positive impact of contact with NGOs could explained by the fact that farmers who have contacts with these extension organizations are likely to hear about improved varieties and thus have more incentive to adopt these new agricultural technologies^49^. The negatively influence of the membership to farmers association and the contact of surveyed farmers with government extensions on the adoption of improved rice varieties could be explained by the frequency of contacts. In addition, as notified by Anik and Salam^54^, farmers who are not satisfied by the services of extension agents will adopt less the improved varieties. In Ghana, Bruce et al*.*^48^ also found a negatively influence of extension services on the adoption of improved rice varieties. The use of fertilizer was also a negative determinant factor of adoption of improved rice varieties in the study area. This is not surprising because, the use of fertilizers is not required to obtain a good yield, when producing some improved rice varieties^55^. These determinants of adoption of improved varieties should be taken in account in the formulation of any transfer policy of improved rice in Republic of Benin.

## Conclusion

For the first time the rice farming systems, the production constraints throughout main rice growing areas and the main factors influencing the adoption of improved rice varieties by Beninese farmers were identified. The results showed that, in the Republic of Benin, there are several types of rice farming system, and most of which are non-mechanized with little use of agricultural inputs, which explains the low yields. The lowland rainfed system and rice monoculture were the dominant cropping patterns. We recommend that, policy initiatives must prioritize formal credit policy for allowing rice farmers to face the various costs related to rice production and purchase farm machinery. Interventions to increase rice yields should target farmers training on rice intercropping practices, irrigated rice system practices, and pest management. The land ownership, crop diversification, production of off-season rice, and contact of farmers with NGOs were identified as affecting positively the adoption of improved rice varieties in the study area. These implies that, extension services (government and NGOs) in charge of diffusion of improved rice varieties to Beninese farmers, should target landowners’ farmers practising off-season rice production, and having in addition to agricultural income, other income from various activities. The negatively influence of membership of farmers’ association and contact with government extension services on the adoption of improved rice varieties must be overcome by strengthening the capacity of extension services and increasing the frequency and quality of trainings and meetings of farmers.

## Data Availability

Raw and treated data generated during study are available from the corresponding author on reasonable request.
